# Complete Heart Block and Persistent Lactic Acidosis as an Initial Presentation of Non-Hodgkin Lymphoma in a Critically Ill Newly Diagnosed AIDS Patient

**DOI:** 10.1155/2014/214970

**Published:** 2014-11-06

**Authors:** Mohsin Ijaz, Hassan Tariq, Masooma Niazi, Dmitry Lvovsky

**Affiliations:** ^1^Division of Pulmonary and Critical Care Medicine, Department of Medicine, Bronx Lebanon Hospital Center, 1650 Selwyn Avenue, Suit No. 12 F, Bronx, NY 10457, USA; ^2^Department of Medicine, Bronx Lebanon Hospital Center, 1650 Selwyn Avenue, Suit No. 10 C, Bronx, NY 10457, USA; ^3^Department of Pathology, Bronx Lebanon Hospital Center, 1650 Selwyn Avenue, Suit No. 10 C, Bronx, NY 10457, USA

## Abstract

A 66-year-old male with newly diagnosed untreated acquired immunodeficiency syndrome (AIDS) presented with chronic nonspecific complaints of weakness, fatigue, myalgia, and weight loss. His initial EKG showed complete heart block necessitating temporary pacemaker placement. He had no previous history of cardiac disease. He was also found to have a persistent lactic acidosis and imaging studies showed abdominal lymphadenopathy. The patient underwent biopsy of these lymph nodes and was found to have diffuse large B-cell lymphoma. The hospital course was complicated by respiratory failure requiring mechanical ventilator support and cardiac arrest. Patient remained critically ill; he was not a candidate for chemotherapy and, after a month of hospitalization, he died. Lactic acidosis and heart block as an initial presentation of non-Hodgkin lymphoma in an AIDS patient are an unusual and unique presentation.

## 1. Introduction

Non-Hodgkin lymphoma (NHL) comprises a diverse group of hematological malignancies. The rate of NHL is estimated to be about 5–20% in human immunodeficiency virus (HIV) infected population, despite the availability of antiretroviral therapy (ART) [1–5]. The clinical presentation range greatly depends upon the subtype of lymphoma and body organ involved and can vary from indolent to very aggressive type. Cardiac infiltration of lymphoma is well recognized and remains asymptomatic in majority of the cases but there are some reports of lymphoma involving the heart presenting with third degree heart block [6–8]. Lactic acidosis is very frequently seen in intensive care unit (ICU) as a cause of anion gap metabolic acidosis and is a significant marker of mortality. Lactic acidosis is associated with a number of clinical entities; however, it is rarely associated with lymphoma, leukemia, and solid organ malignancies. In these conditions the pathogenesis of lactic acidosis remains poorly understood and the management is also a dilemma [9–11].

## 2. Case Presentation

A 66-year-old male was admitted to the ICU with complaints of chronic weakness, fatigue, myalgia, weight loss, and left flank pain. Comorbidities included hypertension, active smoking, and diabetes mellitus. Patient denied nausea, vomiting, diarrhea, hematemesis, melena, hematochezia, chest pain, or any shortness of breath. He had a recent admission due to community acquired pneumonia and was treated with antibiotics. He denied using any illicit drugs or alcohol abuse. He was retired truck driver by profession.

On examination, he was an elderly man with clinical signs of dehydration. Blood pressure was 155/56 mm Hg, heart rate was 67/min, respiratory rate was 16/min, was afebrile, and his oxygen saturation was 99% on room air. He had mild epigastric tenderness without guarding or rigidity. He had bilateral lower extremities chronic skin changes with scaling. The rest of the physical examination was normal.

Significant laboratory data revealed serum sodium 124 mEq/mL, potassium 5.1 mEq/mL, chloride 89 mEq/mL, bicarbonate 15 mEq/mL, blood urea nitrogen 44 mg/dL, creatinine 1.5 mg/dL, glucose 85 mg/dL, calcium 10.3 mg/dL, total protein 7.3 g/dL, and albumin 2.9 g/dL. The rest of the liver function tests were within normal limit. Anemia was with hemoglobin 9.4 g/dL, hematocrit 27%, platelets 558 k/*μ*L, and white blood cells 10.4 k/*μ*L. Arterial blood gas analysis showed pH of 7.36, pCO_2_ of 25 mm Hg, pO_2_ of 90.3 mm Hg, and oxygen saturation of 97% on room air. There was an anion gap of 20, lactic acid of 4.8 mmoles/L, serum lipase of 1540 U/L, amylase 406 unit/L, gamma GT 25 unit/L, INR 1.3, and lactate dehydrogenase (LDH) 950 unit/L; cancer antigen CA 19-9 was 49.9 *μ*/mL, TSH was 2.25 mIU/L, serum acetone was negative, and alcohol level was less than 10 mg/dL. His serial cardiac enzymes were negative and he was ruled out for an acute coronary event.

**Figure 1:**
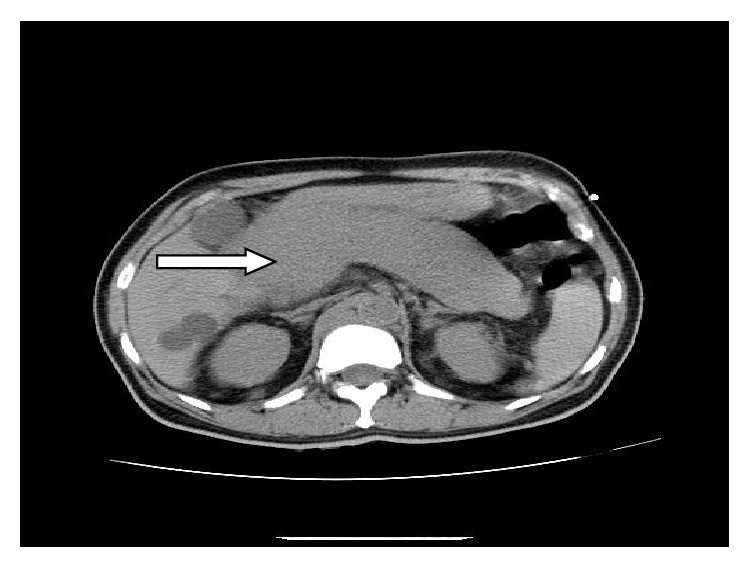
Diffuse enlargement of the pancreas with homogeneous attenuation and without significant peripancreatic inflammation.

**Figure 2:**
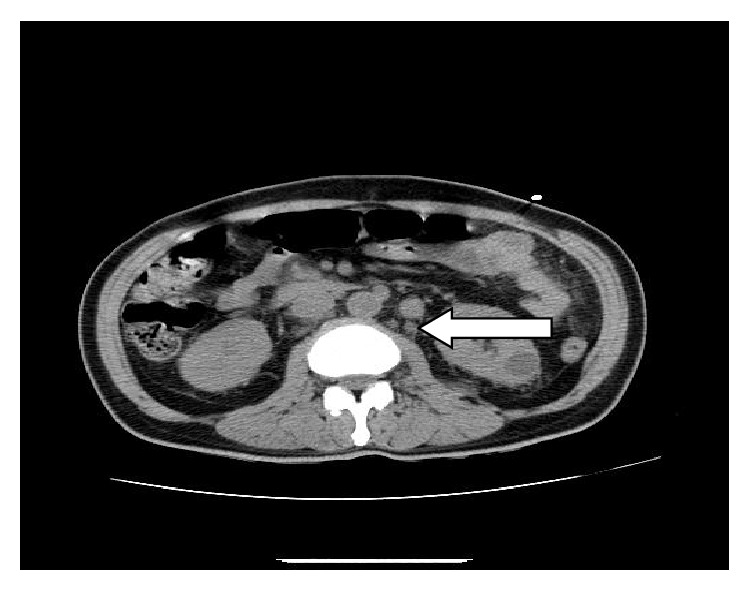
CT of abdomen without contrast showing lymphadenopathy.

Chest X-ray (CXR) revealed bilateral patchy airspace disease. CT of abdomen and pelvis without contrast showed diffuse enlargement of the pancreas with homogeneous attenuation and without significant peripancreatic inflammation (Figure 1) with extensive retroperitoneal and pelvic adenopathy (Figure 2). A para-aortic lymph node measuring 2.1 cm and retrocaval lymph node measuring 1.6 cm were seen. A soft tissue density surrounding the right common iliac vessels likely representing confluent adenopathy was seen. His echocardiogram showed an ejection fraction of 67%; right ventricle systolic pressure was estimated to be 44 mm Hg. Pericardium was normal without any pericardial effusion. There was no significant valvular abnormality.

Initially, the patients EKG showed 2nd degree Mobitz type I block which later progressed to complete heart block. Patient was given atropine without improvement; hence, a temporary pacemaker was inserted. He was started on IV hydration and IV antibiotics and all his electrolyte abnormalities were corrected. His temporary pacemaker lead came out and it was removed. Subsequently he became unresponsive, was orally intubated, had four cardiac arrests, and was successfully resuscitated. Due to persistent pulmonary infiltrates he underwent fiberoptic bronchoscopy with bronchoalveolar lavage (BAL) and transbronchial biopsies which showed evidence of pneumocystis jiroveci pneumonia (PJP). He was started on Bactrim for the treatment of PJP. He tested positive for HIV and was found to have a CD 4 count of 12/uL.

**Figure 3:**
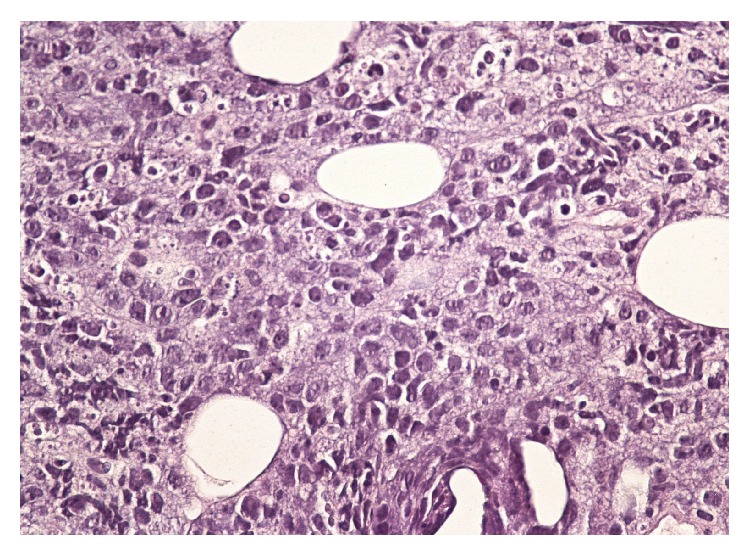
Diffuse large cell lymphoma with atypical lymphocytes, apoptosis, and mitotic figures.

**Figure 4:**
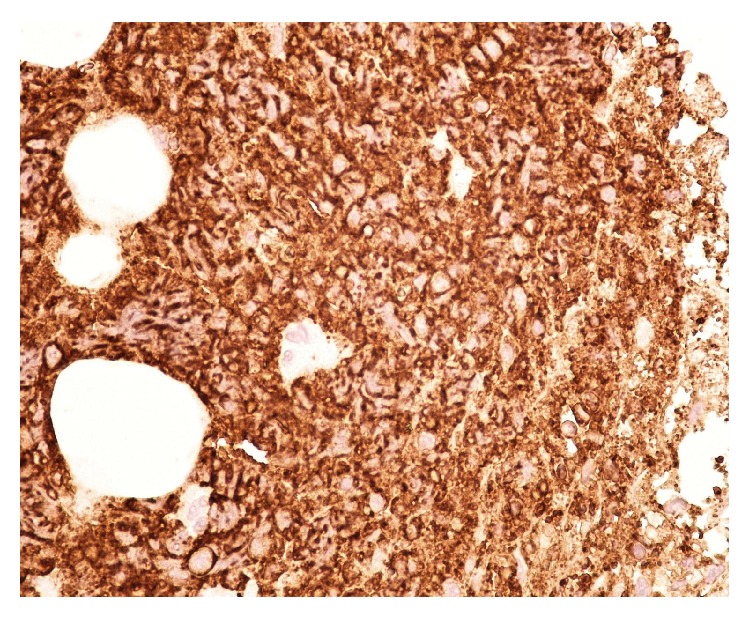
Lymphoma cells are strongly immunoreactive to CD 20.

A temporary pacemaker wire was again placed after ruling out the relevant reversible causes of third degree heart block. A repeat echocardiogram was unchanged from prior one. CT-guided biopsy of the para-aortic lymph nodes was consistent with diffuse large B-cell lymphoma with marked tumor necrosis (Figure 3). Immunoprofile showed that CD 20 (Figure 4), CD 79a, and CD 10 were positive. CD 43, CD 3, CD 7, and BcL were negative. Upper endoscopy for percutaneous endoscopic gastrostomy placement showed gastric polyps which revealed gastric lymphoma on biopsy.

Patient was not a candidate for chemotherapy or ART due to critical condition. Hospital course was complicated by acute respiratory distress syndrome, acute kidney injury, and eventual death a month into his hospital stay.

## 3. Discussion

NHL is the most common form of lymphoma in patients with HIV/AIDS and it is an AIDS defining condition as well. HIV viremia is thought to play a significant role in the pathogenesis of lymphoma [1]. The incidence of NHL has decreased after the advent of ART, however, it has been reported to be 4 to 23 times higher in HIV infected people as compared to those who are not HIV infected [2, 3]. In a large multicenter cohort study in the United States by Gopal et al., there was an increased risk of death among HIV-infected patients with high viral load and NHL, between six months and five years after diagnosis of lymphoma [4]. Advanced stage of extra nodal large B cell lymphoma can involve the heart as part of distant metastasis in 9–24% of cases in autopsy series [5].

Metastatic tumors of the heart are more common than primary cardiac tumors. Lung, breast, melanoma and lymphomas are the most common primary sites for heart metastasis. In an autopsy study, among various cardiac complications of lymphoma the incidence of atrioventricular (AV) block is reported to be about 12%, out of 33 patients with cardiac lymphoma one patient had first degree AV block, two patients had second degree AV block and one patient had third degree AV block [6]. In the same study, patients with lymphoma dying with cardiac involvement revealed that 16% of patients with Hodgkin's disease, 25% with non-Hodgkin's lymphoma, and 33% of cases of mycosis fungoides had cardiac involvement [6]. There have been about 20 reported cases of patients with lymphomas infiltrating the heart presenting with AV block [7], complete heart block was described in a very few cases [6–8]. Sudden appearance of heart block in a patient with lymphoma and no underlying cardiac disease should lead to a suspicion of cardiac involvement. In general, malignant lymphoma which is initially presenting with cardiac symptoms is rarely diagnosed premortally, and has a poor prognosis [8].

Lactic acidosis is a rare complication of lymphomas and its presence is considered an ominous sign [9]. Patients with lymphoma induced lactic acidosis are often critically ill and it is difficult to conclude whether the etiology of lactic acidosis is completely a result of malignancy and not other potential cause such as sepsis [10, 11]. Lactic acidosis secondary to lymphoma is considered a paraneoplastic syndrome [10]. Proposed mechanisms of pathogenesis include increased glycolytic activity causing an increase in lactic acid generation, overexpression of the glycolytic enzyme hexokinase II or increased IGF-binding protein (IGFBP) activity [9, 11]. In highly aggressive tumors, lactate production increases as the tumor outgrows its blood supply resulting in local hypoxia in the absence of any systemic hypoxia or hypoperfusion. The poor prognosis of lactic acidosis in malignancy can be judged from fact that among 29 published cases of lymphoma with lactic acidosis, 25 eventually died [11, 12].

Several imaging modalities, including echocardiography, cardiac CT, MRI and FDG PET may allow an early diagnosis and can suggest the tumor type. Cytology is diagnostic in two-thirds of cases [13]. If pericardial effusion is present, cytological analysis of pericardial fluid may demonstrate malignant lymphocytes [14]. The prognosis of cardiac involvement in lymphoma remains poor due to diagnostic delay and advanced stage of organ infiltration [13].

Systemic chemotherapy appears to be the only effective therapy for cardiac involvement by malignant lymphomas, and patients may require pacemaker for heart blocks [13]. The cyclophosphamide, adriamycin (doxorubicin), vincristine, prednisone (CHOP) regimen was among the first combinations to produce complete response rates and long-term survivors [13]. Complete AV block may be reversible in some patients with metastatic cardiac lymphoma treated by resection of the heart tumor and sequential combination chemotherapy [15, 16]. The addition of rituximab to the CHOP protocol (R-CHOP) increases the overall survival rate. Whenever a complete AV block caused by an infiltration of lymphoma cells occurs, chemotherapy is considered to be the treatment of choice, as well as the implantation of a pacemaker [13].

**Figure 5:**
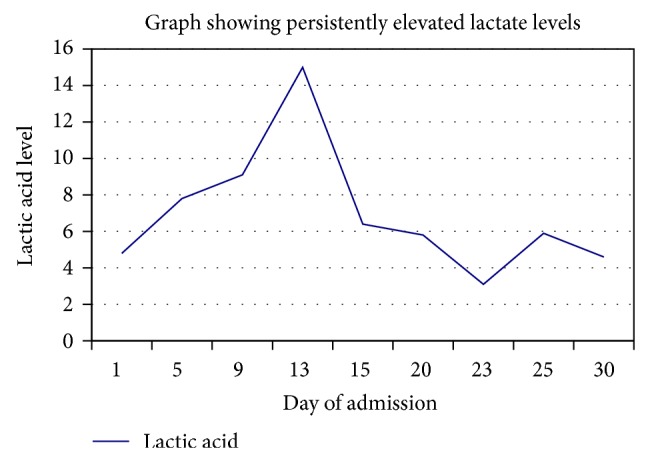
Graph showing persistently elevated lactic acid level.

Considering no previous cardiac disease or invasive procedure, normal echocardiogram, no use of any nodal blocker medication and the presence of persistent lactic acidosis (Figure 5) our suspicion was that complete heart block was most likely from the lymphoma infiltrating the heart. Nevertheless, patients with advanced HIV disease can have various cardiovascular manifestations like cardiomyopathy, myocarditis, pericarditis, endocarditis, valvular disease, pericardial effusion and pulmonary hypertension [17, 18]. Our patient did not have any clinical or echocardiographic abnormality suggesting HIV related cardiac disease.

To the best of our knowledge this case represents the only case in English literature in which a patient with non-Hodgkin's lymphoma presented with both complete heart block and lactic acidosis which individually are rare presentations of NHL which makes this case very unusual and unique.
